# Inequalities in Knowledge About COVID-19 in the Kingdom of Saudi Arabia

**DOI:** 10.3389/fpubh.2021.743520

**Published:** 2021-10-15

**Authors:** Omar Zayyan Alsharqi, Ameerah M. N. Qattan, Noor Alshareef, Gowokani Chijere Chirwa, Mohammed Khaled Al-Hanawi

**Affiliations:** ^1^Department of Health Services and Hospital Administration, Faculty of Economics and Administration, King Abdulaziz University, Jeddah, Saudi Arabia; ^2^Health Economics Research Group, King Abdulaziz University, Jeddah, Saudi Arabia; ^3^Economics Department, Chancellor College, University of Malawi, Zomba, Malawi

**Keywords:** concentration curve, concentration index, COVID-19, inequality, Kingdom of Saudi Arabia (KSA)

## Abstract

**Background:** As the world is still being ravaged by the coronavirus disease 2019 (COVID-19) pandemic, the first line of prevention lies in understanding the causative and preventive factors of the disease. However, given varied socioeconomic circumstances, there may be some inequality in the level of proper knowledge of COVID-19. Despite a proliferation of studies on COVID-19, the extent and prevalence of inequalities in knowledge about COVID-19 in Saudi Arabia are not known. Most related studies have only focused on understanding the determinants of COVID-19 knowledge. Therefore, the aim of this study was to assess the socioeconomic inequalities in knowledge regarding COVID-19 in Saudi Arabia.

**Methods:** Data were extracted from an online cross-sectional self-reported questionnaire conducted on the knowledge about COVID-19 from 3,388 participants. Frequencies and graphs were used to identify the level and distribution of inequality in knowledge about COVID-19. Concentration curves and concentration indices were further used to assess and quantify the income- and education-related inequality in knowledge about COVID-19.

**Results:** The level of COVID-19 knowledge was high among the surveyed sample, although the extent of knowledge varied. The findings further suggest the existence of socioeconomic inequality in obtaining proper knowledge about COVID-19, indicating that inequality in comprehensive knowledge is disproportionately concentrated among the wealthy (concentration index = 0.016; *P* < 0.001) and highly educated individuals (concentration index = 0.003; *P* = 0.029) in Saudi Arabia.

**Conclusions:** There is inequality in the level of knowledge about COVID-19 among the more socioeconomically privileged population of Saudi Arabia. Given that COVID-19 cases ebb and flow in different waves, it is important that proper policies be put in place that will help in improving knowledge among the lower income and less educated individuals, leading to behavior that can help reduce transmission.

## Introduction

Coronavirus disease 2019 (COVID-19) is a respiratory disease triggered by severe acute respiratory syndrome coronavirus 2 (SARS-CoV-2; formerly called 2019-nCoV), which was initially discovered in December 2019 in Wuhan city, Hubei Province, China (1). On March 2020, COVID-19 was affirmed as a pandemic virus-related infection by the World Health Organization (WHO) with the main symptoms identified as fever, fatigue, myalgia, headache, dry cough, shortness of breath, and dyspnea, which may cause acute respiratory distress syndrome and death; moreover, COVID-19 may manifest as skin symptoms such as an erythematous rash, urticaria, and chicken pox–like lesions (2–6).

The COVID-19 pandemic has affected various spheres of life worldwide, with economic, social, and political impacts (7–9). Following the WHO declaration, countries around the globe, including the Kingdom of Saudi Arabia (KSA), have been leaning on response plans to respond to the pandemic and contain transmission of the virus. The KSA put several measures in place to control the spread of COVID-19 in the country since confirmation of its first case on 2 March 2020 (10).

The control measures taken by the government include implementation of a curfew, mass lockdown of cities and people, suspending all inbounds and outbounds flights, transportation restrictions, shutdown of schools and businesses, rapid deployment of testing protocols, and using novel technologies to trace all cases. Additionally, the religious authorities in the country declared introduction of home prayers, banning the five daily prayers in all mosques across the country, which represents an extraordinary step since Saudi Arabian law is founded on Islamic law with the majority of citizens carrying out prayers five times a day in mosques. Moreover, the Umrah was also suspended along with very limited access to the 2020 Hajj (11).

Accordingly, the KSA government established intensive awareness campaigns through the Ministry of Health (MOH) that were distributed on television, and on various social media and mobile phone platforms. Additionally, the MOH produced a guide to COVID-19 to provide residents with facts and precautionary messages that is available in more than 10 languages (10). These unprecedented efforts at all levels and public awareness campaigns led by the government have contributed to raising awareness of the pandemic among citizens, with the aim of limiting the spread of the virus. It has been argued that public adherence to measures and raising awareness of infectious diseases can greatly contribute to tackling pandemics (12, 13).

A recent study carried out to determine the knowledge, attitudes, and practice toward COVID-19 prevention among the general public in the KSA showed that the majority of people surveyed had a high level of knowledge, optimistic attitudes, and safe practices toward COVID-19 (10). Although knowledge regarding COVID-19 may appear to be relatively high in the KSA, it is important to understand if there is any socioeconomic inequality in COVID-19 knowledge and the relative contribution of various factors to this inequality beyond simply identifying the determinants.

Given varied socioeconomic circumstances, there may be some inequality in the level of proper knowledge of COVID-19. Despite a proliferation of studies on COVID-19, the extent and prevalence of inequalities in knowledge about COVID-19 are still not known. Most of the related studies have primarily focused on understanding the determinants of COVID-19 knowledge, attitudes, and perceptions in Saudi Arabia (10, 14, 15), Jordan (16), Egypt (17), Bangladesh (18), and China (19). All of these previous studies found age and education to be among the principal determinants of having proper COVID-19 knowledge, practices, and attitudes. Another study focusing on the potential link between COVID-19 and mental health found that the pandemic appears to be associated with increased stress among healthcare workers in the KSA (20).

As there is a dearth of empirical literature focusing on socioeconomic inequality in COVID-19 knowledge, the aim of this study was to assess the socioeconomic inequality in knowledge regarding COVID-19 in the KSA, based on the use of concentration indices and concentration curves, and identifying the extent to which various factors contribute to any observed inequality in this knowledge. Several factors served as the motivation to perform this study, including established variation in the knowledge levels regarding COVID-19, but without an understanding if socioeconomic inequality contributes to this variation. Proper knowledge regarding an epidemic/pandemic is important in the management of any disease. To our knowledge, this is the first study to assess socioeconomic inequality in knowledge regarding COVID-19 in a quantitative manner using concentration indices and concentration curves.

## Materials and Methods

### Study Design and Sample

The data for this study were extracted from an online cross-sectional self-reported questionnaire that was conducted to investigate the knowledge of the Saudi public toward COVID-19 from 20 March 2020 to 24 March 2020 (10). The online questionnaire was developed according to guidelines for the community of COVID-19, by the Centres for Disease Control and Prevention (CDC) (21). The questionnaire was originally designed in English and translated into Arabic. The questionnaire was translated then back to English to ensure the meaning of the content. The Arabic text was used to administer the study. The survey was administered online to comply with government restrictions put in place to limit human contact so as to minimize COVID-19 transmission. Participants were provided with a questionnaire administered using modern survey techniques, including use of the SurveyMonkey Inc. (San Mateo, CA, USA) distributed to the respondents via Twitter (San Francisco, CA, USA) and WhatsApp Inc. (Mountain View, CA, USA). Participants were recruited using a simplified snowball sampling technique, where the invited participants were requested to pass on the invitations to their contacts. This online survey approach was feasible owing to the high rate of digital adoption in the KSA (4).

According to the latest KSA census, Saudi Arabia has a population of 34,218,169 (22). The representative target sample size needed, to achieve the study objectives and sufficient statistical power, was calculated with a sample size calculator (23). Using a margin of error of +/– 4%, a confidence level of 99%, a 50% response distribution, and 34,218,169 people, the sample size calculator arrived at 1,037 participants. The targeted respondents included being aged 18 years or older and living in the KSA at the time of data collection. Online informed consent was obtained from all participants before proceeding with the questionnaire. A total of 3427 participants completed the questionnaire. After excluding 39 respondents who reported living outside the KSA, the final sample consisted of 3,388 participants.

### Outcome Variable

The dependent variable for this study is the score of the knowledge related to COVID-19. Knowledge of COVID-19 was quantitatively assessed based on the responses to knowledge items in the survey as either true or false, with an additional “don't know” option. Whenever the respondent provided a correct answer, a score of 1 was given, whereas if an incorrect or uncertain (don't know) response was given, a score of 0 was assigned for that question. In total, an aggregate score for knowledge was calculated, which ranged from 0 to 22. A higher score indicates better knowledge of COVID-19, whereas a lower score indicates low knowledge. To assess the internal reliability, Cronbach's alpha was calculated; a Cronbach's alpha value of at least 0.70 demonstrates internal reliability of the instrument (24). Details on the measurement tool can be found elsewhere (10).

### Explanatory Variables

Some explanatory variables were also assessed to place the results in context. Respondents were asked about their socio-demographic characteristics, including their gender, age, marital status, education level, employment status, income level, and region in which they were currently residing. Gender was coded as a binary variable, with a value of 1 assigned for men and 0 for women. The age variable was divided into five categories: 18–29 (reference category), 30–39, 40–49, 50–59, and ≥60 years. This categorization was used to investigate whether there was any difference in the knowledge related to COVID-19 according to age group. With respect to marital status, individuals were assigned a value of 1 if they reported being married and were assigned a value of 0 for unmarried (including single, widowed, and divorced). Education level was categorized as high school or below (reference category), college/university degree, and postgraduate degree. Employment status was classified as government employee (reference category), non-government employee, retired, self-employed, and unemployed. Monthly income (Saudi Riyal, SR 1 = USD 0.27) was grouped into eight categories: < SR 3,000 (reference category), SR 3,000 to <5,000, SR 5,000 to <7,000, SR 7,000 to <10,000, SR 10,000 to <15,000, SR 15,000 to <20,000, SR 20,000 to < 30,000, and SR 30,000 or more. We also controlled for variation among the 13 administrative regions of the KSA: Almadina Almonawra, Albaha, Aljouf/Quriat, Aseer/Bisha, Eastern Region, Haiel, Jazan, Najran, Northern Borders, Qaseem, Riyadh, Tabouk, and Western Region.

### Data Analyses

Various levels of analysis were performed. Firstly, frequencies of individual variables of interest were calculated to understand their distribution. Secondly, to assess socioeconomic inequality, the methodology of Wagstaff et al. (25) was adopted. This includes visualization and estimation of inequalities using the concentration curve and the concentration index.

The concentration curve is a visual approach that plots the cumulative percentage of a health variable on the vertical axis against the cumulative share of that variable in the population (ranked from the lowest to the highest by an indicator of the socio-economic status [SES]) on the horizontal axis. A concentration curve above (below) the line of equality indicates that the proper knowledge related to COVID-19 is concentrated among the poor (rich). The further the concentration curve is away from the line of equality (i.e., the 45-degree line), the greater the degree of inequality.

The concentration index was calculated as twice the area between the concentration curve and the line of equality to quantifies the degree of socioeconomic-related inequality in health or healthcare use (26). The concentration index is defined mathematically as;


(1) $$\begin{array}{r} \text{CI}=\frac{2}{\mu }cov\left(y_{i},r_{i}\right) \end{array}$$

where *yi* is the indicator of COVID-19 knowledge for an individual *i, ri* is the fractional ranking of individuals according to SES and μ is the mean of *yi*. The concentration index can be either negative or positive and ranges between +1 and −1. In this analysis, a negative concentration index indicates that proper knowledge is concentrated among individuals with a relatively low SES, whereas a positive concentration index means that proper COVID-19 knowledge is concentrated among the relatively higher SES. A concentration index of 0 means that no inequality exists in the knowledge of COVID-19.

Moving beyond concentration index calculation, we take a step to understand how each factor contribute to the observed socioeconomic inequality in the knowledge about COVID-19. This is an important analysis for policymakers to pinpoint the variables they will prioritize to reduce the observed socioeconomic inequality. The decomposition method used employed Wagstaff approach (27). This method enables to partition inequality into its contributing factors. To show this, assume that *Y*_*i*_ knowledge of COVID-19 is a linear and additively separable function *X*_*j*_, the vector of covariates is obtained as:


(2) $$\begin{array}{r} Y_{i}=\;\alpha +\beta_{j}X_{ji}+\varepsilon_{i} \end{array}$$

The concentration index can then be expressed as a weighted sum of the aggregated indices of the different explanatory variables in the model for COVID-19 knowledge with respect to the measure of SES (27), as follows:


(3) $$\begin{array}{r} CI=\overset{J}{\underset{j=1}{\sum }}\beta_{j}\frac{{\bar{X}}_{j}}{\mu }CI_{j}+\;\frac{GCI_{\varepsilon }}{\mu } \end{array}$$

Where β_*j*_ represents the partial effect of knowledge determinants, *CI*_*j*_ represents the concentration indices of ${\bar{X}}_{j}$, and *GCI*_ε_ is the generalized concentration index of the error term. Equation 3 illustrates that the contribution of each variable to inequality is based on the interaction between the elasticity of COVID-19 knowledge ($\beta_{j}^{*}\frac{{\bar{X}}_{j}}{\mu }$) with respect to that variable and SES-related inequality in the distribution of the variable. All analyses were conducted using STATA software (StataCorp LP, Texas, USA).

### Ethical Approval

All procedures performed in this study involving human participants complied with institutional and/or national research committee ethical standards, and with the 1964 Helsinki Declaration and subsequent amendments or equivalent ethical standards. This research has been reviewed and given a favorable opinion by King Abdulaziz University. The study was designed and conducted in accordance with the ethical principles established by King Abdulaziz University. Therefore, ethical approval was obtained from the Biomedical Ethics Research Committee, Faculty of Medicine, King Abdulaziz University (Ref-180-20).

## Results

### Social and Demographic Characteristics

Table 1 presents the social and demographic characteristics of the 3,388 participants included in the analysis for this study. The mean COVID-19 knowledge score was 17.96 (*SD* = 2.24, range: 3–22). In terms of gender, 1,966 (58.03%) were women, and 1,422 (41.97%) were men. The predominant age of the respondents was 18–39 years (an aggregate of 57.73%). The number of married participants was 2,149 (63.43%), whereas 1,239 were unmarried (36.57%). With respect to education level, 56.20% of the respondents had a college or university degree. Less than half of the respondents reported being unemployed (31.76%). According to monthly income, 846 (24.97%) respondents were in the lowest group (SR < 3,000), and 246 (7.26%) were in the highest group (SR ≥ 30,000).

**Table 1:** Social and demographic characteristics of study participants.

**Variable**	**Mean**	**SD**	**Min**	**Max**	**N**	**%**
**Knowledge score**	17.96	2.24	3	22		
**Gender**						
Female					1,966	58.03
Male					1,422	41.97
**Age (years)**						
18–29					1,016	29.99
30–39					940	27.74
40–49					692	20.43
50–59					472	13.93
≥60					268	7.91
**Marital status**						
Not married					1,239	36.57
Married					2,149	63.43
**Education**						
High school or below					539	15.91
College/university degree					1,904	56.20
Postgraduate degree					945	27.89
**Work status**						
Government employee					1,320	38.96
Non-government employee					546	16.12
Retired					314	9.27
Self-employed					135	3.98
Unemployed					1,073	31.67
**Monthly income (SR)**						
<3000					846	24.97
3000 to <5000					293	8.65
5000 to <7000					258	7.62
7000 to <10,000					356	10.51
10,000 to <15,000					584	17.24
15,000 to <20,000					472	13.93
20,000 to <30,000					333	9.83
≥30,000					246	7.26
**Region**						
Albaha					15	0.44
Aljouf/Quriat					10	0.30
Almadina Almonawra					147	4.34
Aseer/Bisha					149	4.40
Eastern Region					166	4.90
Haiel					17	0.50
Jazan					19	0.56
Najran					16	0.47
Northern Borders					4	0.12
Qaseem					38	1.12
Riyadh					535	15.79
Tabouk					15	0.44
Western Region					2257	66.62

### Econometric/Statistical Analyses

**Figure 1:**
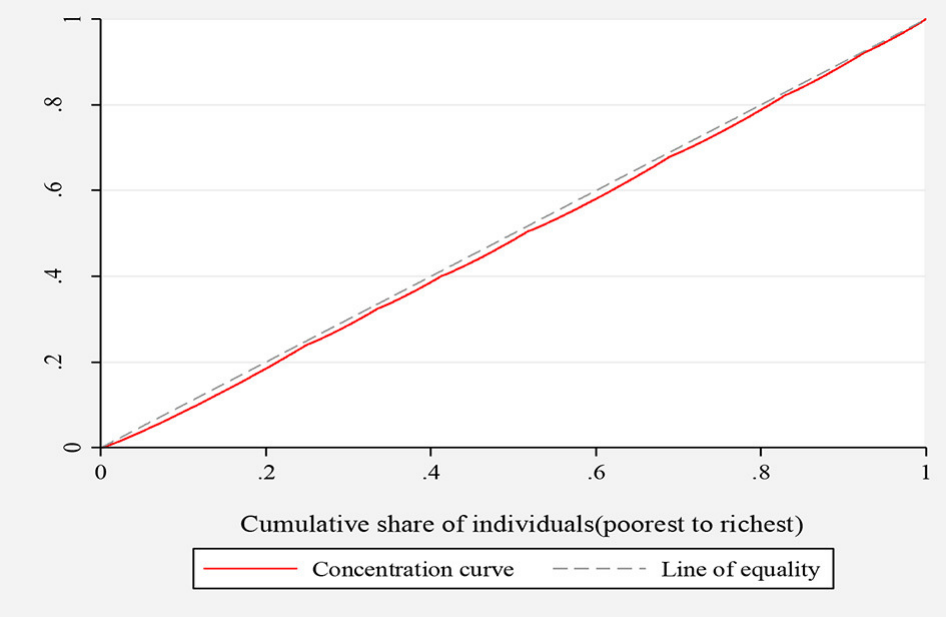
Concentration curve for inequality in COVID-19 knowledge ranked by income.

**Figure 2:**
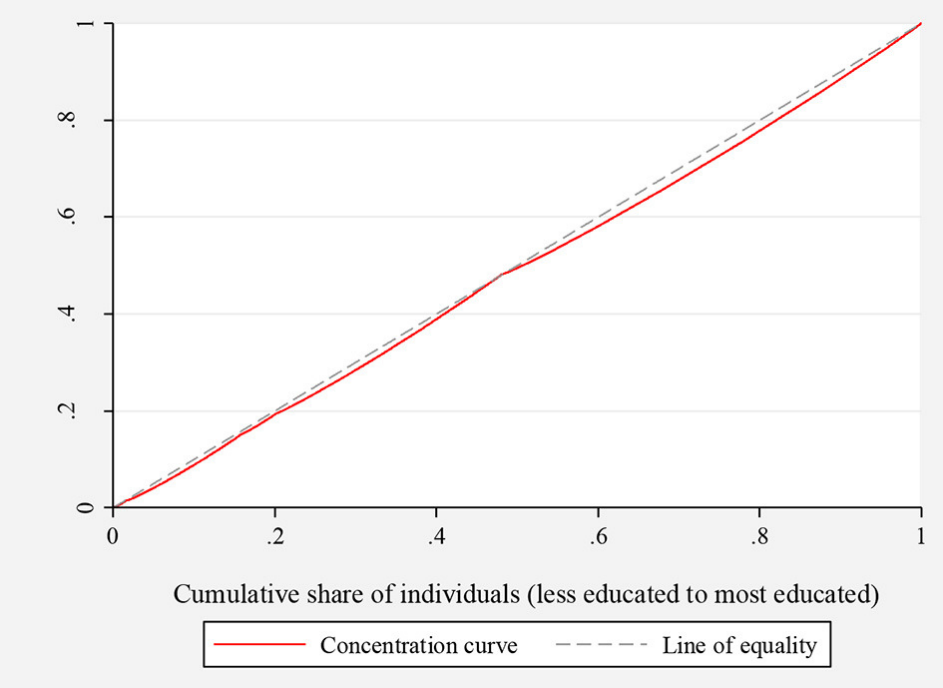
Concentration curve for inequality in COVID-19 knowledge ranked by education.

To assess socio-economic inequalities in the knowledge about COVID-19, concentration indices and concentration curves were used. Figures 1, 2, depict the concentration curves by income and education, respectively. Figure 1 shows that the concentration curve lies to the right-hand side of (below) the line of inequality, indicating the existence of inequality in knowledge regarding COVID-19. Figure 2 shows that the concentration curve was bordered to the right of (below) the line of equality. These analyses thus support that knowledge regarding COVID-19 is disproportionately concentrated among the wealthy and highly educated people in the KSA.

**Figure 3:**
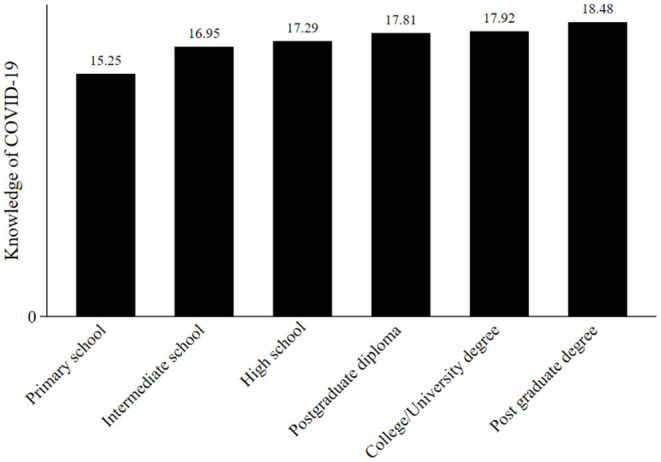
Knowledge of COVID-19 ranked by education level.

The study further assessed the variation in COVID-19 knowledge by education status in further detail. Figure 3 shows that knowledge was higher among highly educated (those with a university degree and above) and was lower among the less educated (those with only a primary school education), indicating a positive trend.

Since the concentration curves do not indicate the magnitude of inequality, we quantified the overall income- and education-related inequality using the concentration indices. The concentration index value for income was significant and positive (0.016, standard deviation = 0.001, *P* < 0.001), indicating that inequality in the knowledge of COVID-19 was concentrated among high-income individuals. Consistently, the concentration index for education was positive and significant (0.003, standard deviation = 0.001, *P* = 0.029), indicating more knowledge of COVID-19 among the most highly educated population in the KSA. These results support the findings from concentration curves presented in Figures 1, 2.

We further assessed the inequality in COVID-19 knowledge among individuals across regions. Although most of the Erreygers index values in each region were positive, there was substantial heterogeneity across regions with respect to ranking according to both education and income. As shown in Table 2, all of the Erreygers indices, except in Northern Borders, were positive; the highest value was obtained in Haiel (0.023; *P* < 0.05), followed by the Qaseem (0.022; *P* < 0.10). Regarding education, the highest value was obtained in Asser/Bisha (0.015; *P* < 0.01), followed by Western region (0.013; *P* < 0.01).

**Table 2:** Concentration indices for the knowledge about COVID-19 by income and education across regions.

	**Income ranking**		**Education ranking**	
**Region**	**Erreygers index**	**Confidence interval**	**Erreygers index**	**Confidence Interval**
AlBaha	0.009	[−0.018, 0.036]	−0.001	[−0.031, 0.029]
AlJouf/Quriat	0.029	[−0.009, 0.067]	0.016	[−0.029, 0.062]
AlMadina Almonawra	0.008	[−0.004, 0.020]	0.013^**^	[0.001, 0.024]
Aseer/Bisha	0.015^***^	[0.005, 0.025]	0.015^***^	[0.006, 0.024]
Eastern Region	0.007	[−0.003, 0.017]	0.005	[−0.007, 0.016]
Haiel	0.023^**^	[0.000, 0.047]	0.014	[0.005, 0.022]
Jazan	0.006	[−0.023, 0.036]	0.010	[−0.020, 0.041]
Najran	0.008	[−0.012, 0.028]	0.011	[−0.008, 0.030]
Northern Borders	−0.020	[−0.078, 0.038]	0.001	[−0.020, 0.022]
Qaseem	0.022^*^	[−0.003, 0.048]	0.016	[−0.013, 0.045]
Riyadh	0.014^***^	[0.007, 0.020]	0.009^***^	[0.001, 0.021]
Tabouk	0.004	[−0.026, 0.034]	0.014	[−0.008, 0.036]
Western region	0.017^***^	[0.014, 0.020]	0.013^***^	[0.010, 0.016]

*** *P < 0.01*,

** *P < 0.05*,

* *P < 0.1*.

Finally, we analyzed how specific factors contribute to the observed inequality in knowledge related to COVID-19. Table 3 shows the results. The analysis showed that much of the inequality is explained by the highest income group SR 20,000 to <30,000 (contribution = 0.003), and SR ≥30,000 (contribution = 0.003). The aggregate contribution of income being about 0.009 (summation of the income contributions). For age, the contributions stand at an aggregate of 0.007. See Table 3 for full details.

**Table 3:** Decomposition of concentration index in COVID-19 knowledge according to demographic variables.

**Variables**	**Coefficient**	**Concentration index**	**Elasticity**	**Contribution**	**%**
**Gender**					
Female (ref)					
Male	−0.316^***^	0.069^***^	−0.025^***^	−0.002^***^	−10.958
	(0.093)	(0.003)	(0.007)	(0.001)	(3.583)
**Age**					
18–29 (ref)					
30–39	0.732^***^	0.063^***^	0.011^***^	0.001^***^	4.488
	(0.131)	(0.017)	(0.002)	(0.000)	(1.663)
40–49	0.620^***^	0.225^***^	0.007^***^	0.002^***^	10.060
	(0.125)	(0.016)	(0.001)	(0.000)	(2.643)
50–59	0.920^***^	0.333^***^	0.007^***^	0.002^***^	15.056
	(0.190)	(0.019)	(0.001)	(0.001)	(3.743)
≥60	0.783^***^	0.464^***^	0.003^***^	0.002^***^	10.148
	(0.200)	(0.024)	(0.001)	(0.000)	(2.882)
**Marital status**					
Unmarried (ref)					
Married	0.166^**^	0.184^***^	0.006^**^	0.001^**^	6.851
	(0.078)	(0.007)	(0.003)	(0.001)	(3.374)
**Education level**					
High school or below (ref)					
College/university degree	0.574^***^	−0.090^***^	0.018^***^	−0.002^***^	−10.259
	(0.137)	(0.009)	(0.004)	(0.000)	(2.908)
Postgraduate degree	0.748^***^	0.378^***^	0.012^***^	0.004^***^	27.883
	(0.136)	(0.012)	(0.002)	(0.001)	(5.919)
**Work status**					
Government employee (ref)					
Non-government employee	0.148	0.028	0.001	0.000	0.236
	(0.131)	(0.020)	(0.001)	(0.000)	(0.300)
Retired	0.180	0.375^***^	0.001	0.000	2.214
	(0.170)	(0.019)	(0.001)	(0.000)	(2.065)
Self-employed	0.116	0.212^***^	0.000	0.000	0.346
	(0.195)	(0.058)	(0.000)	(0.000)	(0.593)
Unemployed	0.183	−0.563^***^	0.003	−0.002	−11.504
	(0.146)	(0.012)	(0.003)	(0.001)	(9.476)
**Monthly income (Saudi Riyal)**					
<3000 (ref)					
3000 to <5000	0.088	−0.414^***^	0.000	−0.000	−1.108
	(0.176)	(0.015)	(0.001)	(0.000)	(2.289)
5000 to <7000	0.037	−0.251^***^	0.000	−0.000	−0.254
	(0.196)	(0.017)	(0.001)	(0.000)	(1.389)
7000 to <10,000	0.210	−0.070^***^	0.001	−0.000	−0.547
	(0.184)	(0.019)	(0.001)	(0.000)	(0.577)
10,000 to <15,000	0.394^**^	0.207^***^	0.004^**^	0.001^**^	4.977
	(0.178)	(0.019)	(0.002)	(0.000)	(2.152)
15,000 to <20,000	0.532^***^	0.519^***^	0.004^***^	0.002^***^	13.593
	(0.198)	(0.016)	(0.002)	(0.001)	(4.944)
20,000 to <30,000	0.765^***^	0.756^***^	0.004^***^	0.003^***^	20.104
	(0.187)	(0.011)	(0.001)	(0.001)	(4.880)
≥30,000	0.779^***^	0.927^***^	0.003^***^	0.003^***^	18.548
	(0.267)	(0.005)	(0.001)	(0.001)	(6.295)
**Region**					
Riyadh (ref)					
Albaha	−0.583	0.173	−0.000	−0.000	−0.105
	(0.627)	(0.165)	(0.000)	(0.000)	(0.186)
Aljoof	0.018	0.013	0.000	0.000	0.003
	(0.416)	(0.043)	(0.001)	(0.000)	(0.270)
Aseer	−0.194	0.065^*^	−0.000	−0.000	−0.197
	(0.425)	(0.036)	(0.001)	(0.000)	(0.407)
Eastern Region	−0.280	0.096^***^	−0.001	−0.000	−0.463
	(0.450)	(0.034)	(0.001)	(0.000)	(0.782)
Haiel	−0.420	0.165	−0.000	−0.000	−0.123
	(0.487)	(0.115)	(0.000)	(0.000)	(0.173)
Jazan	−0.018	−0.155^*^	−0.000	0.000	0.005
	(0.505)	(0.088)	(0.000)	(0.000)	(0.179)
Madenah	−0.410	0.095	−0.000	−0.000	−0.065
	(0.506)	(0.098)	(0.000)	(0.000)	(0.149)
Mekkah	−0.086	0.285^***^	−0.000	−0.000	−0.010
	(0.915)	(0.062)	(0.000)	(0.000)	(0.114)
Najran	−0.247	−0.074	−0.000	0.000	0.072
	(0.423)	(0.079)	(0.000)	(0.000)	(0.184)
Northern Border	−0.306	0.170^***^	−0.003	−0.000	−2.911
	(0.386)	(0.021)	(0.003)	(0.001)	(3.657)
Qassim	0.293	−0.297^**^	0.000	−0.000	−0.136
	(0.562)	(0.133)	(0.000)	(0.000)	(0.320)
Tabuk	−0.337	−0.051^***^	−0.013	0.001	4.059
	(0.380)	(0.006)	(0.014)	(0.001)	(4.399)
**N**	3,388	3,388	3,388	3,388	3,388
**Explained inequality**				0.0150	100
**Overall inequality**				0.0157	
**Residual**				0.0007	

* *p < 0.10*,

** *p < 0.05*,

*** *p < 0.01*.

*Bootstrapped standard errors in blackest*.

## Discussion

To the best of our knowledge, this study provides the first empirical evidence on the existence of socioeconomic inequality in knowledge related to COVID-19 in the KSA. With the spread of COVID-19, numerous countries, including Saudi Arabia, declared a state of emergency, instituting stay-at-home orders, closing schools, and suspending flights. Although these unprecedented policies may help to curb the spread of the virus, having better knowledge impacts how people relate to the disease and behave. Indeed, knowledge has been shown to contribute to an individual's health-related decision-making (28). For instance, in Saudi Arabia, vulnerable groups such as those with a low SES reported lower adoption of preventive measures (29), which may be partly attributed to lower levels of knowledge about the disease. Previous research on infectious diseases has shown that knowledge and beliefs are significant predictors of the adoption of preventive behaviors (30–32). Therefore, the aim of this study was to assess socioeconomic inequalities in COVID-19–related knowledge among the Saudi population during the early stages of the pandemic. Understanding the extent of COVID-19 knowledge among different SES groups can help to enhance the public health emergency response to infectious diseases. This is necessary given that a society's response to an emergency crisis largely depends on meeting the needs of all population subgroups, especially those who are vulnerable and subject to diversity (28).

In this study, knowledge was evaluated by elucidating participants' knowledge of the epidemiological characteristics of the disease and knowledge of actions that can be taken to minimize exposure to COVID-19. The results reveal that the mean COVID-19 knowledge score was 17.96 (*SD* = 2.24, range: 3–22). This indicates that most study participants demonstrated good knowledge about COVID-19. This finding is consistent with findings from other studies conducted in the KSA (14, 33). Nevertheless, inequality in knowledge was observed in this study.

The results of our study showed that income is related to inequalities in knowledge about COVID-19. Knowledge regarding COVID-19 was significantly concentrated among people with higher SES. The significant positive Erreygers concentration index suggests that the richer have a better advantage in terms of knowledge than the poorer. Although these results cannot be directly compared to other studies since this is the first study to use concentration indices to determine inequalities in this regard, the findings closely mimic some related research from Saudi Arabia (10, 14, 15), showing a positive relationship between COVID-19 knowledge and income. Moreover, our findings of high-income earners having more knowledge regarding COVID-19 than low-income earners is in line with other studies conducted in the USA (34), Northern Thailand (35), and Ethiopia (36). Inequality in COVID-19–related knowledge between low- and high-income groups might be due to greater amounts of exposure to misinformation for the former group with greater access to reliable sources of information for the latter group (37).

This study also showed that another SES factor, level of educational attainment, is strongly associated with inequalities in knowledge about COVID-19, which was consistent with an earlier study (38). The results of this study showed that proper knowledge about COVID-19 is concentrated among highly educated individuals. In previous studies, level of education was identified as an important predictor of knowledge for other communicable diseases such as influenza H1N1 (28). Taken together, these findings suggest that an increased level of educational attainment can enhance an individual's readiness to receive and process complex information, even under uncertain conditions that might complicate the message (28). The association between level of education and knowledge about COVID-19 found in this study indicates that in the early stages of the pandemic, communication strategies about the virus did not reach those with lower educational attainment. Although this may have changed later on, these data suggest that public health authorities should consider differences among population subgroups when developing and designing public communication strategies. Tools and strategies testing their effectiveness and evaluating their impact on the population should also be developed.

Moreover, regional inequalities were also found. The income-based concentration indices and education-based concentration indices were positive and significant for some regions. This indicates inequality related COVID-19 across regions. For example, the income-based concentration indices were positive and significant for Aseer, Haiel, Riyadh and the Western Region. This indicates that residents of Haiel, Riyadh and the Western Region had more knowledge than those of other regions in the country. Although the reason behind this regional variability is unclear, differences in knowledge between different regions in the country can be attributed to the average income between regions. Moreover, this may be due to variation in the circumstances surrounding the opportunities brought about by the respective provincial economies.

After decomposition, of the Erreygers index, we found that a large share of the inequality is explained by the income inequality. Not only that, age, also played a significant role, in addition to the education related inequalities. All these put together may be assumed to affect inequality and incomes probably being the transmission route through which they are playing. Since these factors always reinforce each other, they may indeed push the inequality through some sort of a vicious circle. However, despite these being the major factors, it doesn't rule out the impact of the other factors, although they are somehow smaller in terms of the contribution.

In terms of policy, we may suggest that the authorities should devise strategies that, take into account, differences in income and education to ensure widespread outreach. For example, they should enhance the dissemination of more messages on social media, since most people may have access to that. Moreover, they may consider the use of community outreach by health personnel, where they would educate people in their local environment, which would cover issues to do with low income and less education. This would be the case since the health personnel would be tasked, in accordance with the community, they would be paying a visit at a specific point in time.

In spite of the above findings and recommendations, this study is not without limitations. The construction of our dependent variable is based on a well-known study (10) but is also different from the variables used in other studies (39, 40). Therefore, the difference in the type of measure used may have an impact on assessing the inequality for the variable. Moreover, the nature of our analysis does not permit making inferences related to causality. Endogeneity may be present because the variable of interest is constructed from recall, which may suffer from reporting bias. We could not find an appropriate instrument variable from the data that could appropriately deal with the potential endogeneity issue. Because of the highlighted shortfalls, the implication for future research is that using quasi-experimental identification methods such as instrumental variable matching or synthetic cohort analysis (41, 42) may be better to control for reverse causality. Furthermore, it may also be important to use different measures of the knowledge score that would enable comparisons of the outcome. Lastly, our study did not use a complex sample survey that appropriately stratifies the KSA population. In the absence of a complex sample survey this result may only apply to the sample collected.

## Conclusions

In conclusion, inequality in knowledge related to COVID-19 in the KSA highlights the importance of ensuring the dissemination of health information to all subgroups of the population, regardless of their social class or individual and geographic backgrounds. Our findings suggest that public health authorities may need to tailor more creative communication strategies and select different communication channels that can be used to diffuse health information to all social groups. As a way forward, in terms of directions for further research, it may be important to use a Sharpley decomposition to understand the drivers of this inequality. Our current data do not allow for such a complicated analysis.

## Data Availability Statement

The datasets generated and/or analyzed during the current study are not publicly available due to privacy and confidentiality agreements as well as other restrictions but are available from the corresponding author (MA-H) on reasonable request.

## Ethics Statement

This research has been reviewed and given a favorable opinion by King Abdulaziz University. The study was designed and conducted in accordance with the ethical principles established by King Abdulaziz University. Therefore, ethical approval was obtained from the Biomedical Ethics Research Committee, Faculty of Medicine, King Abdulaziz University (Ref-180-20).

## Author Contributions

All authors listed have made a substantial, direct and intellectual contribution to the work, and approved it for publication.

## Funding

This project was funded by the Deanship of Scientific Research (DSR) at King Abdulaziz University, Jeddah, under Grant No. PH: 7-120-1442. The funders had no role in study design, data collection and analysis, decision to publish, or preparation of the manuscript.

## Conflict of Interest

The authors declare that the research was conducted in the absence of any commercial or financial relationships that could be construed as a potential conflict of interest.

## Publisher's Note

All claims expressed in this article are solely those of the authors and do not necessarily represent those of their affiliated organizations, or those of the publisher, the editors and the reviewers. Any product that may be evaluated in this article, or claim that may be made by its manufacturer, is not guaranteed or endorsed by the publisher.
